# The complete mitochondrial genome of *Cochliobolus miyabeanus* (Dothideomycetes, Pleosporaceae) causing brown spot disease of rice

**DOI:** 10.1080/23802359.2019.1660273

**Published:** 2019-09-02

**Authors:** Gang Deng, Qian Zou, Yue Chen, Lingxian Wang, Ge Huang, Yongzhen Cui, Mingliang Ding, Yuanbing Wang

**Affiliations:** a School of Agriculture, Yunnan University, Kunming, China;; b The Institute of Food Crops, Yunnan Academy of Agriculture Sciences, Kunming, China;; c Biotechnology and Germplasm Resources Institute, Yunnan Academy of Agricultural Sciences, Kunming, China;; d Yunnan Herbal Laboratory, Institute of Herb Biotic Resources, School of Life Sciences, Yunnan University, Kunming, China;; e The Research Center of Cordyceps Development and Utilization of Kunming, Yunnan Herbal Biotech Co. Ltd., Kunming, China

**Keywords:** *Cochliobolus miyabeanus*, mitochondrial genome, phylogenetic analysis

## Abstract

*Cochliobolus miyabeanus* is known as a significant causal agent in relation to brown spot disease of rice and causes significant yield losses. In the present study, the complete mitochondrial genome was determined using next-generation sequencing technology. This complete mitogenome is a circular molecule of 124,887 bp in length. It contains 13 conserved protein-coding genes, 21 transfer RNA genes, 2 ribosomal RNA genes and 9 open reading frames. The overall base composition of *C. miyabeanus* is 35.4% A, 34.4% T, 14.4% C, 15.8% G, with a CG content of 30.2%. Phylogenetic analysis based on concatenated protein genes from 15 taxa within Pezizomycotina showed that *C. miyabeanus* is closely related to *Bipolaris cookei* in the family Pleosporaceae (Dothideomycetes, Pleosporales). This work would facilitate the understanding of systematics and evolutionary biology of phytopathogenic fungi.

Rice (*Oryza sativa* L.) is the staple food for nearly two-thirds of the world’s population and is considered one of the most important crops by the contribution to human diet and huge value of production (Bockelman et al. 2003). However, brown spot caused by *Cochliobolus miyabeanus* (S. Ito & Kurib.) Drechsler ex Dastur (anamorph: *Bipolaris oryzae*) is a prevalent fungal disease of rice with a worldwide distribution, especially in the rice-growing countries of Asia, America, and Africa. Rice is susceptible to this spot diseases which adversely affect grain yield and quality, causing significant economic losses (Nazari et al. 2015). In 1942, brown spot caused yield losses of 90% and brought famine in Bengal (Ghoze et al. 1960), while caused 20, 25, and 43% yield losses in South Africa, India, and Nigeria, respectively (Padmanabhan 1963; Aluko 1975). For *C. miyabeanus*, population structure, genetic diversity and phylogenetic relationships with other related plant pathogens have been investigated (Manamgoda et al. 2012; Nazari et al. 2015; Ahmadpour et al. 2018). However, little is known about its mitochondrial genome (mitogenome). This study aims to report the complete mitogenome of *C. miyabeanus* and reveal its phylogenetic relationship with other related species based on mitochondrial concatenated protein sequences.

*Cochliobolus miyabeanus* strain ATCC 44560 was firstly isolated from rice in Taiwan and deposited at American Type Culture Collection. Pure cultures of this strain on PDA were used to extract total genomic DNA using MiniBEST Universal Genomic DNA Extraction Kit (TaKaRa, China). The whole-genome sequencing was performed on the Illumina sequencing platform (HiSeq PE150) with standard procedures. Mitogenomic sequences of the high-quality reads were assembled by SPAdes 3.9.0 with default parameter (Bankevich et al. 2012). Mitogenome was annotated using MFannot tool (http://megasun.bch.umontreal.ca/cgi-bin/mfannot/mfannotInterface.pl) and ARWEN web server. The mitogenomic circular map of *C. miyabeanus* was depicted using Organellar Genome DRAW tool (Lohse et al. 2007).

The complete mitogenome of *C. miyabeanus* is a closed loop and consists of 124,887 bp in length. Its annotated mitogenome was submitted to GenBank under accession no. MN148434. It contains 13 conserved protein-coding genes with many intron sequences, 21 transfer RNA genes, 2 ribosomal RNA genes and 9 open reading frames (ORFs). The 13 conserved protein-coding genes include seven subunits of NADH dehydrogenase (nad1, nad2, nad3, nad4, nad4L, nad5, and nad6), three subunits of cytochrome c oxidase (cox1, cox2, and cox3), one subunit of ATPase (atp6), ubichinol cytochrome c reductase (cob), and ribosomal protein (rps3). A large number of ORFs with unknown functions consist of ORF349, ORF421, ORF363, ORF444, ORF376, ORF360, ORF510, ORF577, and ORF1138. The overall base composition is as follows: 35.4% A, 34.4% T, 14.4% C, 15.8% G, with a CG content of 30.2%.

To determine the phylogenetic position of *C. miyabeanus*, mitogenomic sequences of 14 taxa within Pezizomycotina were downloaded from NCBI. Mitogenomic sequences of *C. miyabeanus* and other taxa were aligned using the programme HomBlocks (Bi et al. 2018). Phylogenetic analysis based on the 14 concatenated protein sequences of 15 taxa were performed using Maximum Likelihood (ML) method with RaxML 7.0.3 (Stamatakis 2006). The best model GTR + I was selected for ML analysis with 500 rapid bootstrap replicates. As shown in Figure 1, phylogenetic tree reveals the topological structure of 15 taxa within Pezizomycotina. *Cochliobolus miyabeanus* is clustered with *B. cookei* (Sacc.) Shoemaker in the family Pleosporaceae (Dothideomycetes, Pleosporales) and is statistically well supported by the bootstrap proportions of ML analysis (ML-BP= 100%).

**Figure 1.**
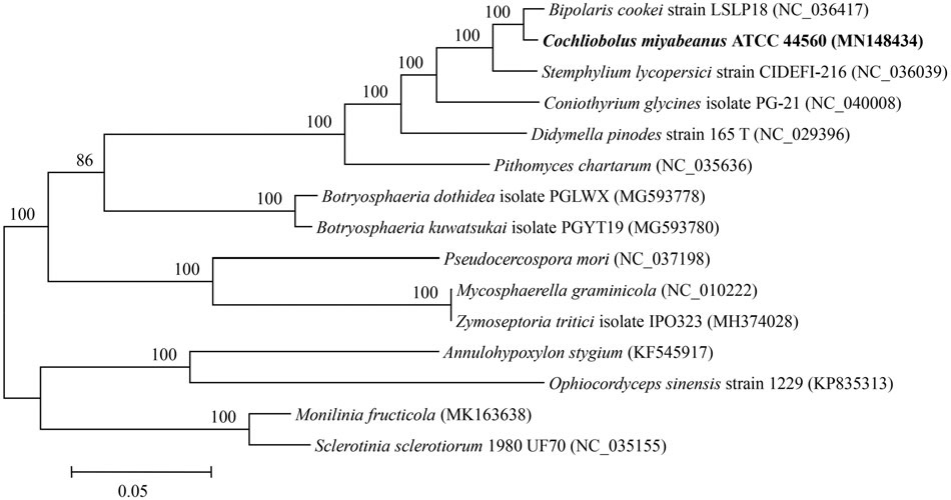
Phylogenetic relationships among 15 taxa of Pezizomycotina were determined by HomBlocks, resulting in 7529 bp collinear alignment of each mitochondrial genome. The phylogenetic tree was constructed by maximum likelihood (ML) method. The bootstraps are shown above internodes.
